# Mixed-phase structural characterization of urchin-like CuO/Cu₂O nanostructures

**DOI:** 10.1186/s42649-026-00140-y

**Published:** 2026-06-10

**Authors:** Hye Seong Jang, Gyeong Hee Ryu

**Affiliations:** 1 Department of Materials Engineering and Convergence Technology, Gyeongsang National University, Jinju, 52828 Republic of Korea; 2 School of Materials Science and Engineering, Gyeongsang National University, Jinju, 52828 Republic of Korea

**Keywords:** Mixed-phase nanostructure, Surfactant-assisted method, Urchin-like

## Abstract

In this study, urchin-like CuO/Cu₂O mixed-phase nanostructures were synthesized via a simple surfactant-assisted approach, where the surfactant regulated the nucleation and anisotropic growth, leading to a hierarchical morphology with radially arranged nanoneedles. X-ray diffraction (XRD) and X-ray photoelectron spectroscopy (XPS) analyses verified the coexistence of CuO and Cu₂O phases within individual microspheres. High-resolution transmission electron microscopy (HRTEM) revealed well-defined lattice fringes assigned to Cu₂O, while XRD, XPS, SAED, and EDS analyses collectively suggest the coexistence of CuO and Cu₂O phases within the hierarchical microspheres. Energy-dispersive spectroscopy (EDS) mapping and line-scan profiles indicated a gradual compositional variation of copper and oxygen from the core to the surface region. Furthermore, in situ electron-beam irradiation demonstrated structural densification of the urchin-like architecture, providing insight into its structural stability. These results offer fundamental understanding of mixed-phase copper oxide formation and highlight their potential for high-surface-area functional materials.

## Introduction

Copper oxides, specifically cupric oxide (CuO) and cuprous oxide (Cu_2_O), have long been established as fundamental p-type semiconductor materials in the fields of electronics (Al-Jawhari 2015; Maeng et al. 2016) and energy conversion (Al-Jawhari 2015; Chen 2013). In the current era of materials science, where sustainability and eco-friendliness are paramount (Horváth 2018; Chen et al. 2020), copper-based oxides are witnessing a significant resurgence. Their earth-abundance, non-toxicity, and low production costs make them ideal candidates to replace rare or precious metals in next-generation functional devices. However, conventional copper oxides in bulk or simple nanoparticle forms often suffer from limited specific surface areas and rapid charge carrier recombination (Lu et al. 2024), which hinder their performance in high-efficiency applications.

To overcome these intrinsic limitations, recent research has focused on two primary strategies: the design of hierarchical 3D geometries (Cao et al. 2007) and the engineering of mixed phase (Dey et al. 2021) heterostructures. Among various architectures, the urchin-like morphology characterized by a dense core with radially aligned nanoneedles offers a remarkably high specific surface area (Xu et al. 2009; Hong et al. 2009). This hierarchical structure not only provides a vast number of active sites for surface reactions but also creates open diffusion channels that facilitate rapid mass transport. Furthermore, the construction of a mixed-phase CuO/Cu_2_O system creates a p-p heterojunction (Yang et al. 2016). The resulting internal electric field at the interface is expected to promote efficient charge separation and transport, which could potentially enhance the sensitivity and catalytic activity compared to single-phase counterparts.

In this study, we report the successful synthesis of urchin-like CuO/Cu_2_O mixed-phase microspheres via a facile surfactant-assisted method (Jang et al. 2023; 2025a; 2025b; Lee et al. 2024a; 2024b). The surfactant plays a dual role: it acts as a capping agent to direct anisotropic growth into radial nanoneedles and ensures morphological uniformity. We present a comprehensive structural characterization using XRD, XPS, and HRTEM to elucidate the crystallinity and chemical states of the heterostructure. A key highlight of this work is the precise analysis of the spatial elemental gradient from the core to the edge of the microspheres, as revealed by EDS mapping, which provides insights into the phase evolution during growth. Furthermore, we investigated the structural durability and real-time transformation of these hierarchical architectures under electron-beam (E-beam) irradiation. By providing a structural and phase-controlled framework for potentially improving the performance of traditional copper oxides, this research offers a fundamental basis for designing high-efficiency, low-cost functional materials for future energy and sensing applications.

## Method

The CuO/Cu_2_O nanostructures were synthesized via a surfactant-assisted method. Initially, two separate 5 mM aqueous solutions were prepared: one containing copper (II) nitrate hydrate (Cu(NO_3_)_2_H_2_O) and the other containing hexamethylenetetramine (HMTA). Both solutions were dispersed using a sonicator for 10 min to ensure complete dissolution. Subsequently, the 5 mM copper nitrate solution and the 5 mM HMTA solution were mixed in a 210 mL Glasslock container. To direct the morphological growth, a surfactant mixture was prepared by blending 0.0024 g of sodium hexadecyl sulfate C_16_H_33_NaO_4_, 95%, Tokyo Chemical Industry), 10 mL of chloroform (CHCl_3_, 99.5%, Sigma-Aldrich), and 0.0024 g of oleylamine. This surfactant solution was added dropwise into the aqueous mixture in the Glasslock container and thoroughly dispersed. The resulting mixture was exposed to ambient air for 30 min. For the thermal treatment, the container was placed on a hot plate) and maintained at 80℃ for 3 h. After the reaction, the container was removed and allowed to cool at room temperature for 10 min. For structural and morphological characterization, the synthesized metal oxide nanostructures were transferred onto SiO_2_-coated Silicon (Si) wafers.

### Material characterization

The morphological and chemical properties of the Urchin-shape were characterized using SEM (COXEM, JEOL JSM-7610F), XRD (Bruker D8 Advance A25 Plus), and XPS (Thermo Fisher K-alpha). Raman spectra were obtained using a Raman spectrometer (Nanophoton, RAMANtouch), and UV–vis absorption spectra were recorded using a UV–vis spectrophotometer (Cary 60). XPS data were acquired from urchin-shape transferred onto a 50 nm thick platinum coated Si substrate to minimize interference from the native oxide layer of the Si substrate. Peak deconvolution was performed after background subtraction using the Shirley method. The specimens were further analyzed using Cs-corrected TEM (JEMARM200F, NEO ARM with STEM-Cs) and the TF30ST device (Thermo Fisher) at 200 and 300 kV, respectively, at the Center for Research Facilities, Gyeongsang National University. For the in situ electron-beam irradiation experiment, TEM observations were performed at an accelerating voltage of 200 kV under continuous electron-beam irradiation. Sequential TEM images were acquired at approximately 10 s intervals. The beam current, absolute electron dose, and dose rate were not directly calibrated during the experiment; therefore, the beam-induced morphological evolution was interpreted qualitatively rather than as a quantitative dose-dependent transformation.

## Results and discussion

**Fig. 1:**
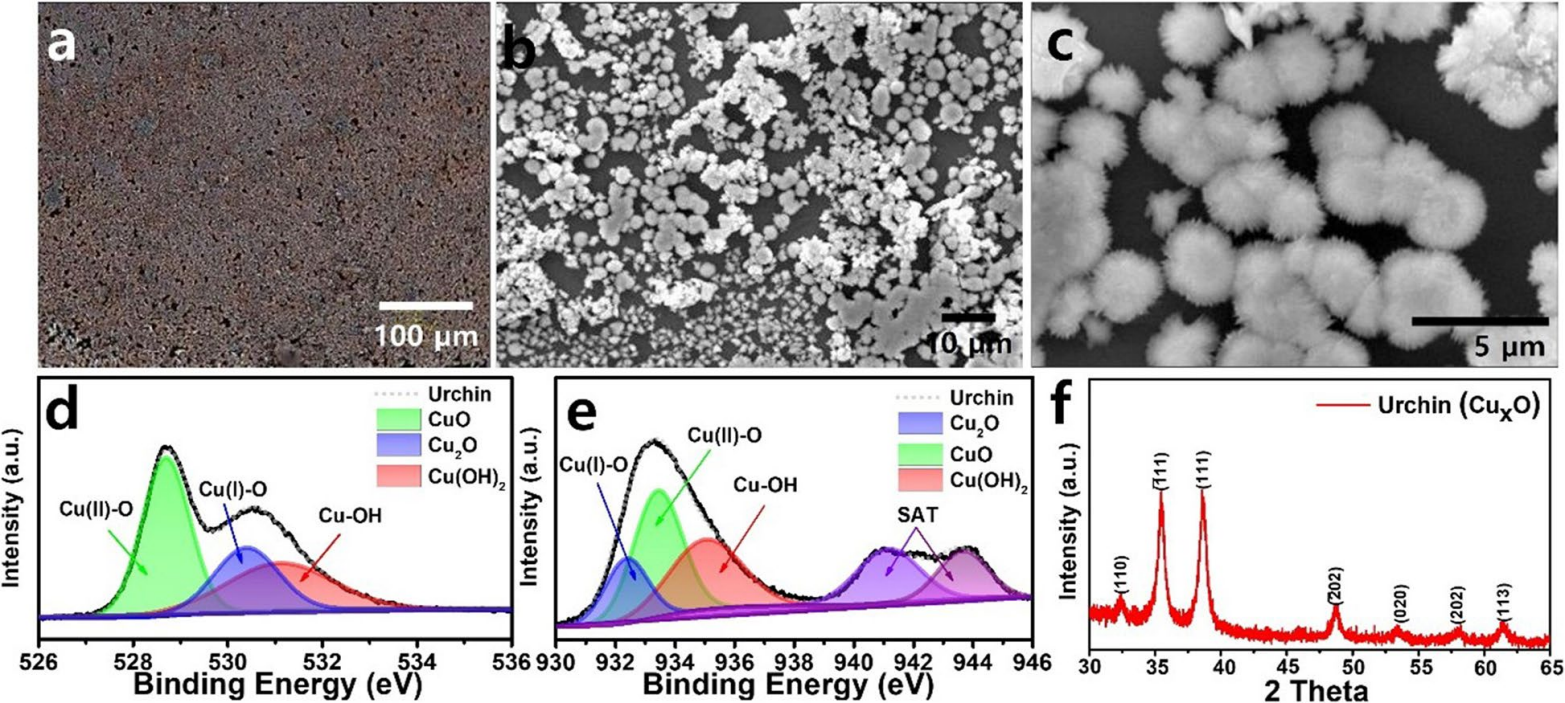
Morphological and structural characterization of urchin-like CuₓO. **a** Optical microscopy image of the synthesized material. **b**, **c** SEM images showing the urchin-like morphology composed of radially grown nanostructures. **d** O1s XPS spectrum with deconvoluted peaks corresponding to Cu(II)–O, Cu(I)–O, and Cu–OH species. **e** Cu 2p XPS spectrum showing Cu₂O, CuO, and Cu(OH)₂ components with satellite features. (f) XRD pattern of the synthesized Urchin like

The morphology and phase purity of the synthesized nanostructures were first examined using SEM, XPS, and XRD (Fig. 1). Figure 1a–c shows the SEM images at various magnifications, revealing the successful formation of highly uniform, urchin-like microspheres. At low magnification (Fig. 1a), the large-scale uniformity of the product is evident. Higher magnification images (Fig. 1b-c) show that each microsphere, with an average diameter of approximately 2–3 μm, consists of numerous radially aligned nanoneedles, creating a hierarchical architecture with a high surface-to-volume ratio.

To investigate the chemical composition and oxidation states, X-ray Photoelectron Spectroscopy (XPS) was conducted. The Cu 2p spectrum (Fig. 1e) exhibits two main peaks corresponding to Cu 2p _3/2_ and Cu 2p _1/2_. Notably, the presence of strong satellite peaks (indicated by red arrows) at higher binding energies is a characteristic feature of Cu^2+^ in CuO. Furthermore, the deconvolution of the Cu peaks and the O 1 s spectrum (Fig. 1d) confirms the coexistence of Cu^+^ and Cu^2+^ species, they were assigned to Cu–OH at 531.1 eV, CuO at 530.38 eV, and Cu₂O at 529.68 eV, respectively (Yang et al. 2010; Biesinger et al. 2011; Svintsitskiy et al. 2011). indicating the formation of a mixed-phase CuO/Cu_2_O heterostructure.

The crystalline structure was further validated by X-ray Diffraction (XRD) (Fig. 1f). The diffraction patterns exhibit distinct peaks that can be indexed to both the monoclinic CuO (tenorite) and cubic Cu_2_O (cuprite) phases. These peaks were assigned to (110), (−111), (111), (202), (020), and (113) (Gurav et al. 2013; Lu et al. 2004; Ni et al. 2009; Sahooli et al. 2012; Rodriguez et al. 2003). The relatively broad peaks suggest the nanocrystalline nature of the building blocks (nanoneedles) that comprise the urchin-like spheres. No other impurity peaks were detected, confirming the high purity of the synthesized CuO/Cu_2_O mixed-phase.

**Fig. 2:**
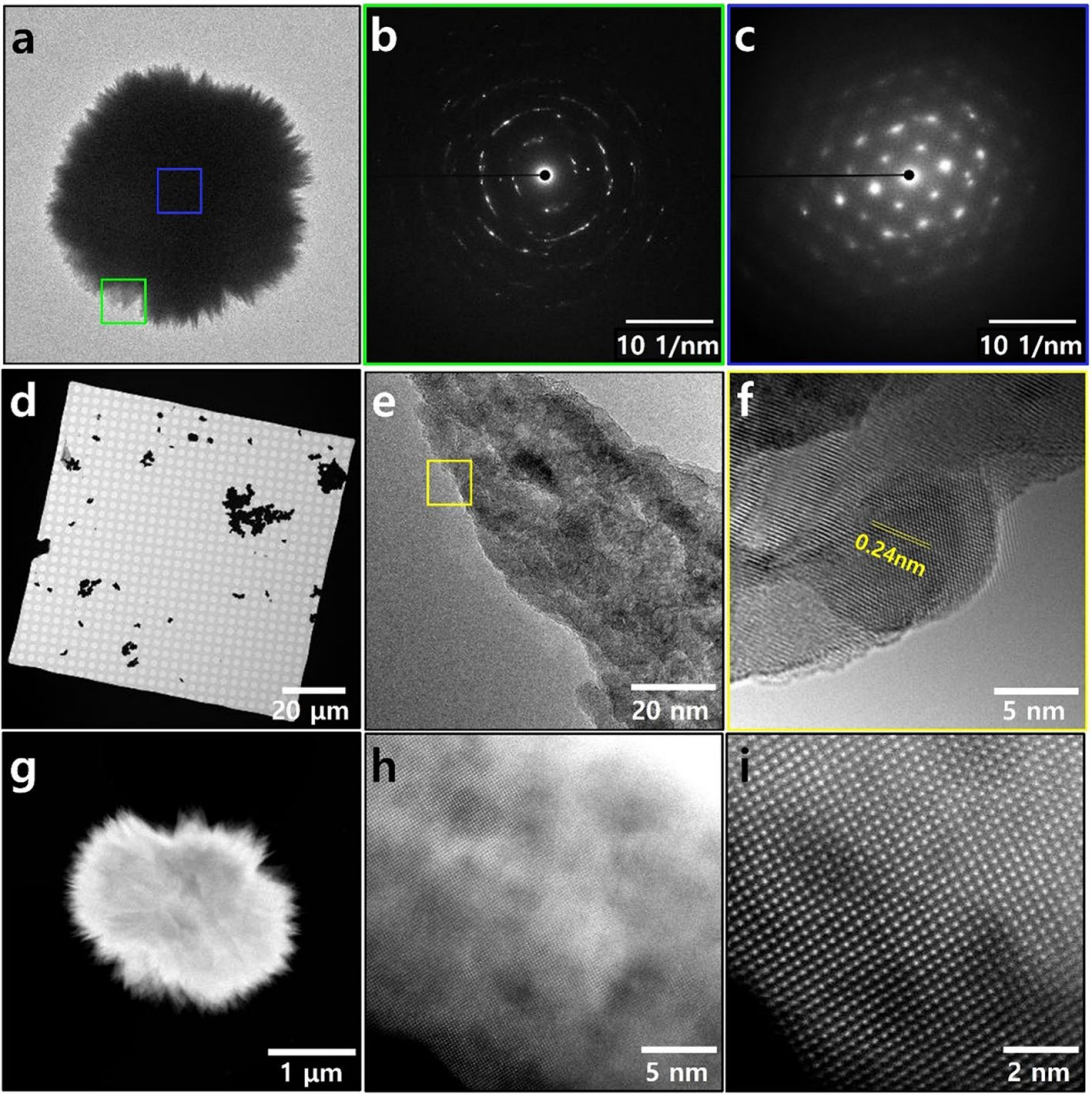
TEM characterization of urchin-like CuO/Cu₂O microspheres. **a** Low-magnification TEM image of a single urchin-like particle. **b** SAED pattern obtained from the edge region marked by the green box in (**a**). **c** SAED pattern from the core region marked by the blue box in (**a**). **d** Low-magnification TEM image showing dispersed particles on the TEM grid. **e** TEM image of a nanoneedle extracted from the urchin-like structure. **f** HRTEM image showing lattice fringes with an interplanar spacing of ~ 0.24 nm. **g** HAADF-STEM image of the urchin-like particle. **h**, **i** Atomic-resolution STEM images revealing the crystalline lattice structure

As shown in Fig. 2, high-resolution transmission electron microscopy (HRTEM) and scanning transmission electron microscopy (STEM) were employed to further investigate the hierarchical structure and internal crystallinity of the urchin-like CuO/Cu₂O microspheres. The low-magnification TEM image (Fig. 2a) clearly visualizes the three-dimensional urchin-like morphology, consisting of a densely packed central core with radially protruding nanoneedles, with an overall diameter of approximately 2 μm. This morphology suggests that the surfactant played an important role in regulating the anisotropic growth of the needle-like structures.

To resolve the crystallographic differences within the hierarchical architecture, selected-area electron diffraction (SAED) analysis was performed on two distinct regions the peripheral needles (green box) and the central core (blue box). The SAED pattern obtained from the edge region (Fig. 2b) exhibits a series of well-defined diffraction rings, characteristic of a polycrystalline structure. These diffraction rings can be indexed to the crystallographic planes of CuO. In contrast, the SAED pattern collected from the central region (Fig. 2c) displays a more discrete spot-like arrangement superimposed on the rings, suggesting higher crystallinity or the presence of aggregated nanocrystalline domains compared to the outer needle region. This difference may originate from the longer effective reaction time experienced by the core during the early stage of the Surfactant assistent process.

Further insight into the atomic structure was obtained from high-resolution TEM imaging. As shown in Fig. 2f, the nanoneedles exhibit well-resolved lattice fringes, indicating high crystallinity. The measured interplanar spacing of approximately 0.24 nm corresponds well to the (111) plane of cubic Cu₂O. This observation provides direct real-space evidence that copper oxide phase constitute the primary structural component of the hierarchical needle. Moreover, the continuity of these lattice fringes over relatively large regions suggests that the material maintains high crystallinity with minimal structural defects, such as dislocations or stacking faults, despite the rapid growth induced by HMTA and the surfactant.

The structural integrity of the microspheres was further confirmed by high-angle annular dark-field scanning transmission electron microscopy (HAADF-STEM), as shown in Fig. 2g–i. The Z-contrast imaging in HAADF mode allows clearer differentiation of atomic density within the nanostructure. Atomic-resolution STEM images (Fig. 2h and i) reveal a periodic arrangement of copper atoms, confirming the well-defined lattice structure of the copper oxide system. In addition, the absence of a significant amorphous layer on the needle surface indicates that the surfactant-assisted synthesis effectively stabilizes the crystalline facets without leaving excessive organic residues.

**Fig. 3:**
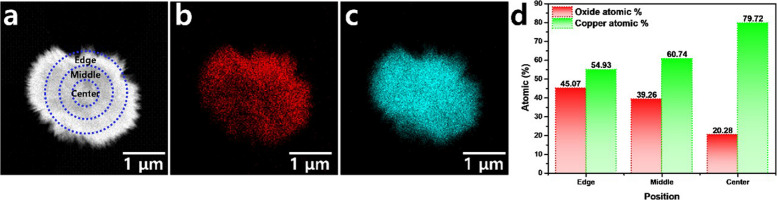
STEM–EDS elemental mapping analysis of the urchin-like CuO/Cu₂O microsphere. **a** HAADF-STEM image with the regions of interest (edge, middle, and center) indicated. **b** Oxygen elemental mapping. **c** Copper elemental mapping. **d** Quantitative atomic percentage of oxygen and copper at different positions (edge, middle, and center)

To further elucidate the internal chemical distribution and the formation mechanism of the urchin-like CuO/Cu2O heterostructure, Energy Dispersive X-ray Spectroscopy (EDS) mapping and quantitative point analysis were performed (Fig. 3). The HAADF-STEM image and corresponding elemental maps for copper (cyan) and oxygen (red) demonstrate that both elements are distributed throughout the entire hierarchical architecture. However, a closer inspection of the relative intensities suggests a non-uniform oxidation state across the microsphere.

As summarized in the quantitative bar graph (Fig. 3, right panel), the atomic percentages of copper and oxygen were measured at three distinct radial positions: the Center, Middle, and Edge. A significant compositional gradient was observed At the Center The copper content reached its maximum at 79.72 at%, with a corresponding oxygen content of only 20.28 at%. This high Cu/O ratio suggests that the core is predominantly composed of Cu-rich species, which is consistent with the presence of Cu_2_O or metallic Cu clusters as inferred from other structural analyses. At the Edge: In stark contrast, the oxygen content increased to 45.07 at%, while the copper content decreased to 54.93 at%. This ratio is much closer to the stoichiometric values of CuO, suggesting that the radially grown nanoneedles are more highly oxidized. This spatial gradient provides crucial evidence for a core-first, oxidation-later growth mechanism. During the initial stages of synthesis, Cu-rich seeds appear to form the dense core.

As the reaction progresses and nanoneedles elongate via the surfactant-assisted template effect, the increased surface area of these needles facilitates more extensive oxidation through contact with the aqueous HMTA environment. Consequently, the resulting structure is a spatially controlled heterostructure featuring a Cu_2_O-rich core and a CuO-rich shell/needle layer. Such a gradient could be beneficial for potential electronic applications, as it may facilitate the creation of a built-in potential that could assist the migration of charge carriers.

**Fig. 4:**
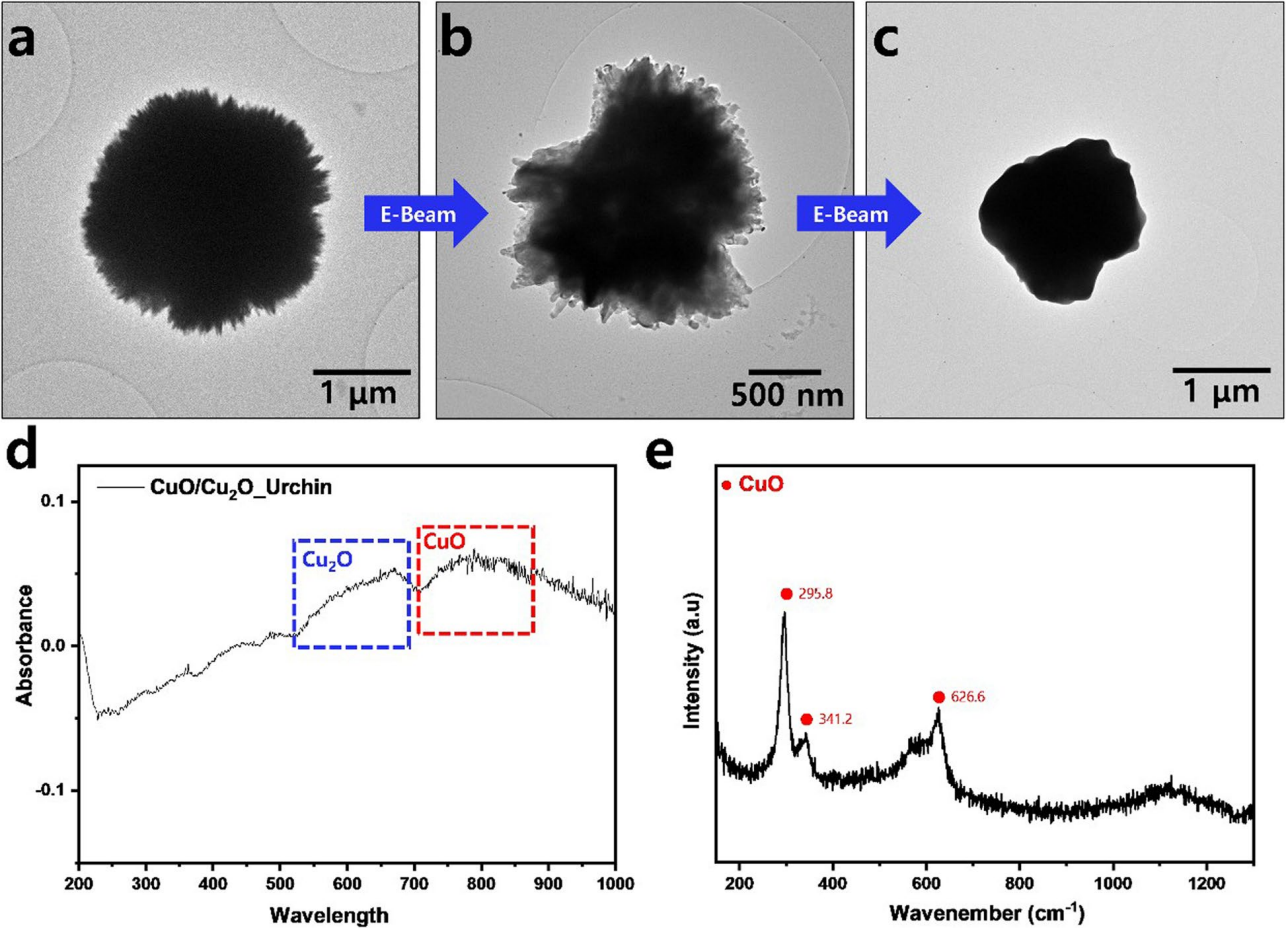
**a**–**c** In situ TEM images showing the qualitative beam-induced structural evolution of an urchin-like CuO/Cu₂O microsphere under continuous electron-beam irradiation at 200 kV. Sequential images were acquired at approximately 10 s intervals. Because the beam current, absolute electron dose, and dose rate were not directly calibrated, the in situ sequence is interpreted qualitatively rather than as a quantitative dose-dependent transformation. **d** UV–Vis absorption spectrum of the synthesized CuO/Cu₂O sample. **e** Raman spectrum showing characteristic CuO-related vibrational modes

To evaluate the structural stability of the urchin-like CuO/Cu₂O nanostructures under high-energy conditions, in situ TEM observations were performed at an accelerating voltage of 200 kV under continuous electron-beam (E-beam) irradiation (Fig. 4a–c). Sequential TEM images were acquired at approximately 10 s intervals. Because the beam current, absolute electron dose, and dose rate were not directly calibrated during the experiment, the observed morphological evolution is discussed qualitatively rather than as a quantitative dose-dependent transformation. Initially, the CuO/Cu₂O microsphere exhibited a well-defined urchin-like morphology composed of radially aligned nanoneedles (Fig. 4a). However, under prolonged E-beam exposure, the hierarchical structure underwent a gradual morphological transformation. The outer nanoneedles progressively retracted and merged toward the central core (Fig. 4b), eventually resulting in the collapse of the hierarchical architecture and the formation of a dense and compact particle (Fig. 4c).

This structural evolution can be attributed to the high surface energy of the nanoneedle morphology. Under E-beam irradiation, surface atoms gain sufficient mobility to migrate, leading to surface reconstruction and a reduction in the total surface energy. In addition, inelastic electron scattering may induce localized heating and radiolytic effects within the CuO/Cu₂O lattice, potentially causing oxygen loss or partial phase transformation. The sequential TEM images also reveal a noticeable volume shrinkage, indicating that the internal porosity generated during synthesis is gradually eliminated through a densification process.

The optical properties of the synthesized CuO/Cu₂O structures were further investigated using UV–Vis absorption spectroscopy (Fig. 4d). The spectrum exhibits broad absorption extending throughout the visible region, which is characteristic of copper oxide (Zhu et al. 2017; Li et al. 2017; El-Trass et al. 2012). This behavior arises from the coexistence of CuO and Cu₂O phases with different band-gap energies. Cu₂O typically has a band gap of ~ 2.0–2.2 eV, while CuO possesses a narrower band gap of ~ 1.2–1.5 eV (Du et al. 2024; Hesabizadeh et al. 2021; Wong et al. 2016), resulting in enhanced visible-light absorption.

To further examine the phase composition, Raman spectroscopy was performed (Fig. 4e). The Raman spectrum exhibits characteristic peaks at 295.75, 341.24, and 626.6 cm⁻^1^, which are mainly consistent with Raman-active modes of monoclinic CuO. Specifically, the peaks at 295.75 cm⁻^1^ and 341.24 cm⁻^1^ are assigned to the A_g and B_g vibrational modes, respectively, whereas the peak at 656.6 cm⁻^1^ is assigned to a CuO-related high-frequency vibrational mode associated with Cu–O lattice vibrations (Guha et al. 1991; Hagemann et al. 1990; Lopez et al. 2021). In contrast, a distinct Cu₂O Raman signature is not clearly resolved in the present spectrum, possibly due to weak intensity, peak broadening, or overlap with the dominant CuO-related modes. Therefore, the Raman result primarily supports the presence of crystalline CuO within the hierarchical structure, rather than independently confirming both CuO and Cu₂O phases.

Overall, the combined structural and spectroscopic analyses indicate that the synthesized material contains mixed CuO/Cu₂O phases, as supported collectively by XRD, XPS, SAED, EDS, and Raman analyses. Among these techniques, Raman spectroscopy mainly supports the CuO component, while the Cu₂O contribution is inferred from the complementary characterization results. The material also exhibits strong optical absorption in the visible region. Although the urchin-like morphology provides a high surface area, the in situ TEM results reveal that the hierarchical architecture is metastable under high-energy irradiation, undergoing gradual densification through nanostructure sintering.

## Conclusions

In this study, mixed-phase CuO/Cu₂O hierarchical microspheres with an urchin-like morphology were successfully synthesized through a surfactant-assisted growth process. Electron microscopy revealed that the microspheres consisted of dense cores and radially aligned nanoneedles, forming a uniform three-dimensional hierarchical structure. Structural analyses confirmed the coexistence of CuO and Cu₂O phases within the same microsphere. UV–Vis and Raman spectroscopy further supported the formation of the mixed copper oxide system, showing broad visible-light absorption and characteristic vibrational modes, indicating the potential for functional applications based on its structural properties and phase coexistence. In situ TEM observations under continuous electron-beam irradiation showed gradual collapse of the nanoneedle structure and transformation into a denser particle due to atomic migration and structural densification, suggesting the metastable nature of the hierarchical architecture under high-energy irradiation.

## Data Availability

The datasets used and/or analysed during the current study are available from the corresponding author on reasonable request.

## Abbreviations

TEM: Transmission Electron Microscopy
SEM: Scanning Electron Microscopy
XRD: X-ray Diffraction
XPS: X-ray Photoelectron Spectroscopy
UV-vis: Ultraviolet-Visible Spectroscopy
E-beam: Electron Beam
3D: Three-Dimensional
HRTEM: High-resolution transmission electron microscopy
EDS: Energy-dispersive X-ray spectroscopy
HMTA: Hexamethylenetetramine
HAADF-STEM: High-Angle Annular Dark-Field Scanning Transmission Electron Microscopy
